# Laser-based technique for controlled damage of mesenchymal cell spheroids: a first step in studying reparation *in vitro*

**DOI:** 10.1242/bio.017145

**Published:** 2016-06-22

**Authors:** N. V. Kosheleva, I. V. Ilina, I. M. Zurina, A. E. Roskova, A. A. Gorkun, A. V. Ovchinnikov, M. B. Agranat, I. N. Saburina

**Affiliations:** 1 FSBSI Institute of General Pathology and Pathophysiology, 8 Baltiyskaya St, Moscow 125315, Russian Federation; 2 Faculty of Biology, Lomonosov Moscow State University, 12-1 Leninskie Gory, Moscow 119234, Russian Federation; 3 Joint Institute for High Temperatures of the Russian Academy of Sciences, 13 Bld 2, Izhorskaya St., Moscow 125412, Russian Federation; 4 Russian Medical Academy of Postgraduate Education, 2/1 Barrikadnaya St., Moscow 123995, Russian Federation

**Keywords:** Spheroids, Multipotent mesenchymal stromal cells, Reparation, Laser microsurgery, Nanosecond laser scalpel

## Abstract

Modern techniques of laser microsurgery of cell spheroids were used to develop a new simple reproducible model for studying repair and regeneration *in vitro*. Nanosecond laser pulses (wavelength 355 nm, frequency 100 Hz, pulse duration 2 ns) were applied to perform a microdissection of the outer and the inner zones of human bone marrow multipotent mesenchymal stromal cells (BM MMSC) spheroids. To achieve effective dissection and preservation of spheroid viability, the energy of laser pulses was optimized and adjusted in the range 7-9 μJ. After microdissection, the edges of the wound surface opened and the angular opening reached a value of more than 180°. The destruction of the initial spheroid structure was observed in the wound area, with surviving cells changing their shape into a round one. Partial restoration of a spheroid form took place in the first six hours. The complete structure restoration accompanying the reparative processes occurred gradually over seven days due to remodelling of surviving cells.

## Abstract (precis)

**Summary:** The technique of precise nanosecond laser microsurgery of mesenchymal cell spheroids was used to develop a new simple reproducible model for studying repair and regeneration *in vitro*.

## INTRODUCTION

Regeneration is the restoration of the lost parts of the body, which can occur after injury at different levels of the organization: i.e. cellular, tissue, organ, structure or whole-body. There are several basic strategies for regeneration: remodelling of the remaining undamaged tissue in the absence of cell proliferation (morphallaxis); replacing the structure due to proliferation of a resident stem cells pool, as well as trans- and de-differentiated cells (epimorphosis); or, a combination of these two processes (Alvarado and Tsonis, 2006). Despite the conservatism of general mechanisms of regeneration in evolution, mammals have almost lost the ability to fully restore vast areas of the body after injury (Bely, 2010; Bely and Nyberg, 2010).

In modern biology, for the model investigation of repair and regeneration, researchers are actively moving from two-dimensional (2D) to three-dimensional (3D) conditions (Haycock, 2011). Multicellular organisms have a 3D organisation, in which cells interact with other cells and an extracellular matrix to form a complex set of contacts, thereby creating a unique microenvironment. Behaviour, proliferative activity and physiological properties of the cells in 3D systems *in vitro*, in contrast to 2D monolayer, are similar to *in vivo* conditions (Cukierman et al., 2001). Various 3D cell cultures are now used to simulate tissue cytoarchitectonics and to create a model system that would resemble the structure of native tissue (Schmeichel and Bissell, 2003; Takezawa et al., 1992). In these 3D cultures, cells grow, interacting with the environment (other cells, extracellular matrix and the external environment) in all three dimensions (Antoni et al., 2015; Koch et al., 2012). In recent years, 3D cultures have been widely used for monitoring cell proliferation, analysis of viability, morphology and differentiation of cells in response to various stimuli, as well as studying cell-cell interactions, migration and invasion of tumour cells into the surrounding tissues (Kubatiev et al., 2015; Pampaloni et al., 2007).

Cellular spheroids are one of the most common options in *ex vivo* studies using 3D cell cultures, along with explant cultures, cells on microcarriers and tissue-engineered systems. They represent 3D spherical cell clusters, which self-organize due to natural adhesive properties. In spheroids obtained from single cell suspension, they not only make intercellular contacts but also contacts with newly synthesized extracellular matrix, thereby forming a structure, the organization of which resembles the organization of tissues *in vivo* (Lin and Chang, 2008). Many cell types have a natural tendency to aggregate; therefore, spheroids can be obtained from one or more cell types (Haycock, 2011). In particular, in their work, V.S. Repin and co-authors (2014) reveal a consistent pattern in the formation of spheroids from cells of epithelial and mesenchymal phenotypes.

The field of application of cellular spheroids is constantly expanding. For example, studying the mechanisms of wound healing is one of the challenging issues nowadays. Using *in vitro* monolayer cultures as a model system allows for studying only specific parameters of cell behaviour (rate of migration and proliferation, extracellular matrix synthesis), but does not allow for evaluating the contribution of intercellular interactions as well as interactions of cells with the extracellular matrix. Hence, similar studies are now performed mainly *in vivo* or by using organotypic explant cultures *ex vivo* (Antoni et al., 2015; Gottrup et al., 2000). Nevertheless, the search for simple reproducible model systems for studying mechanisms of regeneration, problems of the fibrotic and non-fibrotic wound healing continues. Repair of cellular spheroids after damaging effects could be one such model.

It has been established that if the diameter of the spheroid exceeds 200-250 μm, an oxygen concentration gradient appears, with a minimum at the centre of the spheroid (Curcio et al., 2007; Griffith, and Swartz, 2006; Acker et al., 1987). In addition, large spheroids accumulate carbon dioxide and cell waste products, resulting in the formation of necrosis in the centre of the spheroid. Therefore, to ensure proper diffusion of nutrients to the central region, we used cellular spheroids with diameters no more than 200 μm. Given the relatively small size of cellular spheroids, the development of a repair model on this object requires the use of the most advanced technologies. For this study, we chose to use the technique of laser microsurgery to simulate spheroid injury.

In current clinical practice and biology, laser microdissectors based on pulsed lasers have been widely used (Magidson et al., 2007). Using this technique makes microsurgery possible not only at tissue, but also at the cellular and even subcellular levels. Femtosecond laser sources are regarded to be the most promising. They provide high spatial and temporal resolution, and have greater penetration depth, which is extremely important, particularly for affecting interstitial structures. Femtosecond laser systems have been shown to be useful and successful in different fields: for dissection of the nuclei in fixed cells, actin filaments (Heisterkamp et al., 2005; Shen et al., 2005) and chromosomes (König et al., 2001; Uchugonova et al., 2012), as well as separating single living cells from the group (Kohli et al., 2005) and inactivation of cell organelles, such as mitochondria (Watanabe et al., 2004; Shen et al., 2005). These systems allow for not only the formation of incisions on cell membrane (Kohli et al., 2005), but also solving the problem of selective delivery of extracellular substances into cells (optoinjection and transfection) by using short-lived laser perforation of cell membrane (Il'ina et al., 2013, 2014; Baumgart et al., 2008; Uchugonova et al., 2008;. Stevenson et al., 2006). It should also be noted that, in recent years, the possibility of successful microsurgery of intracellular structures (individual centrioles, cytoskeletal elements, spindle microtubules) has been demonstrated by using nanosecond (Magidson et al., 2007; Khodjakov et al., 2004) and picosecond laser systems (Colombelli et al., 2005; Botvinick et al., 2004), with an accuracy comparable to that of femtosecond systems (Sacconi et al., 2005).

In addition to 2D structures (cell monolayers), laser microsurgery techniques have been tested on 3D objects – embryos of different organisms. In particular, a nanosecond laser dissector was used for creating a hole in oocyte zona pellucida for the further *in vitro* fertilization or polar body biopsy (Clement-Sengewald et al., 1996). Later, a laser dissector based on the femtosecond laser was successfully used for solving the problems of assisted hatching and dissection of trophectoderm cells during biopsy at the later stages of pre-implantation development (Ilina et al., 2016, 2015). In addition, picosecond and femtosecond laser pulses were successfully used in performing non-contact fusion and inactivation/enucleation of blastomeres in mouse (Karmenyan et al., 2009) and porcine (Kuetemeyer et al., 2010) embryos, as well as for studying the modulation of morphogenetic movements in *Drosophila melanogaster* embryos (Supatto et al., 2005). Despite the fact that embryos are 3D objects, they serve as convenient models only for a limited number of problems. Meanwhile, cellular spheroids, as a model object, allow us to bridge the gap between living tissue and 2D structures *in vitro*, creating prerequisites for the development of models of laser microsurgery based on cellular spheroids.

However, despite the interest of scientists regarding spheroids and widespread application of lasers in biology and medicine, there are currently only a few studies in which laser technologies are used for studying spheroids. These works are concerned with techniques of spheroid visualization and, in particular, methods of light-sheet-based fluorescence microscopy (Pampaloni et al., 2013; Bruns et al., 2012), two-photon microscopy for studying matrix-guided mechanisms of cell invasion (Ilina et al., 2011), or visualization of spheroids differentiation (König et al., 2011). According to our data, the only study in which the laser was applied directly to impact and locally damage the spheroid was found in the work of Uchugonova and co-authors (2007), who demonstrated the possibility of irreversible optical damaging of single cells, which migrated from the spheroid, while maintaining the viability of surrounding non-irradiated cells. Such a technique of selective laser destruction (knockout) can be used for ‘optical cleaning’ of cell cultures. In turn, the study of the characteristics of the spheroid repair process after large-scale ‘extended’ injuries has not been conducted to date.

The aim of this study, then, was to develop a reproducible model of cellular spheroids injury using laser microsurgery techniques and to investigate the repair process after laser impact. It was decided to use a nanosecond laser dissector to simulate spheroids damage. The energy of the laser pulses were optimized such that the dissection was carried out only in the specified area of the spheroid and, therefore, would not compromise the viability of the spheroid in general. The study was conducted on human bone marrow multipotent mesenchymal stromal cells (BM MMSC).

## RESULTS

The cells in obtained BM MMSC primary culture under 2D conditions successfully formed a monolayer, maintained a high proliferative potential of up to the fourth passage, and had a characteristic spindle-shaped form. After four passages, BM MMSCs expressed characteristic mesenchymal markers (CD105, CD90 and CD29), while they almost did not express haematopoietic and lymphocyte markers CD45, CD34, CD14, CD11b, CD19 (Table 1).

**Table 1.**
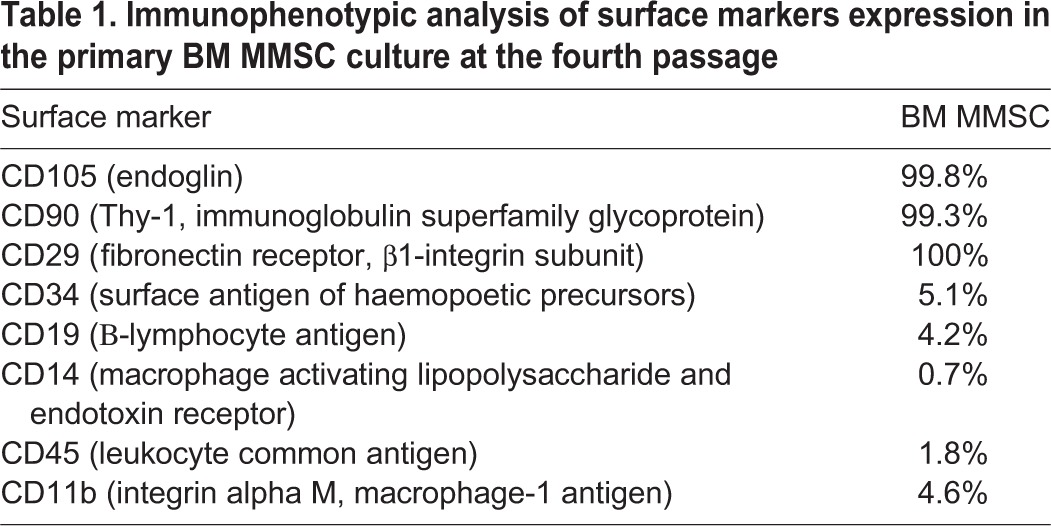
**Immunophenotypic analysis of surface markers expression in the primary BM MMSC culture at the fourth passage**

**Fig. 1.**
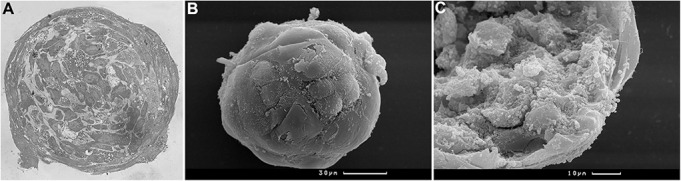
**BM MMSC spheroid structure.** (A) Compact 7-day spheroid has two different regions: two to four layers of elongated surface cells and an internal core consisting of polygonal irregular cells. (B) General view of surface cells. (С) Spheroid fracture displaying imbricated cells of the surface region and the loose inner zone of polygonal cells. A, semi-fine section, stained with methylene green, light microscopy; B,C, scanning electron microscopy.

Under non-adhesive 3D conditions, BM MMSC suspension successfully formed compact viable spheroids in seven days. Histological analysis revealed two distinct regions in compact spheroids: two to four layers of elongated surface cells and an internal core consisting of polygonal irregular cells. Imbricated cells of the surface region were in close contact with each other, whereas the inner zone was loose, and polygonal cells were separated by the extracellular matrix (Fig. 1).

### Injuring BM MMSC 3D cultures with nanosecond laser scalpel

The parameters of the laser radiation and, in particular, the energy of laser pulses were optimized to perform an effective microsurgery of selected regions of the surface and internal areas of the BM MMSC spheroid. The main ‘surgical’ mechanism causing tissue dissection after application of nanosecond laser pulses is the formation of plasma and optical-induced breakdown (Genc et al., 2014; Rau et al., 2006; Vogel et al., 1996). In their work, Genc and colleagues (2014) considered two modes of optical-induced breakdown formation, depending on the energy of laser pulses. In the first case, the energy of pulses was slightly higher than the threshold energy, while optical-induced breakdown was accompanied by the formation of cavitation bubbles the size of a few micrometres. Their emergence has led to visible damage to the irradiated tissue; but, due to the small size of cavitation bubbles, it became possible to perform localized microsurgery of the selected object. The second mode occurred after a considerable excess of pulse energy threshold, characterized by the visible plasma luminescence and formation of large cavitation bubbles (50-100 μm). When applied to spheroids, this mode was highly undesirable, as the precise dissection became practically impossible. The formation of large cavitation bubbles led to a massive and irreversible damage to the whole object.

**Fig. 2.**
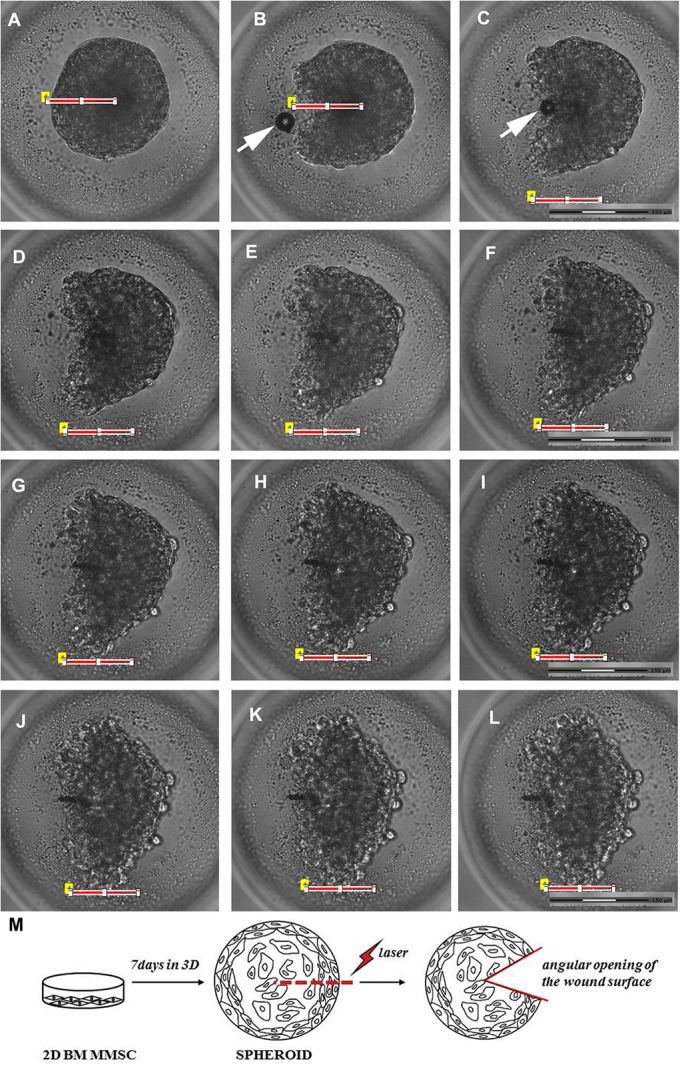
**Live time-lapse microscopy during the first five minutes after the exposure of BM MMSC spheroids to nanosecond laser scalpel impact.** (A) General view of spheroid before laser irradiation. (B) The moment of spheroid injury. (C-L) Spontaneous disclosure of the wound edges, the increasing angular opening of the wound surface: C, 30; D, 40; E, 60; F, 80; G, 100; H, 130; I, 150; J, 200; K, 250; L, 300 s after the start of microdissection. (M) Short scheme of spheroid obtainment and laser exposure. The red line indicates the chosen straight-line path of irradiation. White arrows indicate cavitation bubbles. A-L, light microscopy, phase contrast. Scale bar: 150 μm.

In our experiments we determined, that the threshold energy of laser pulses, required for dissection to start, lay in the range of 6.2-6.6 μJ (the speed of motorized stage movement along *xy* axis was selected to be 120 µm/s). However, the quality of dissection was very sensitive to spheroid's size and heterogeneity of agarose plate thickness, where spheroids were placed. Application of laser pulses with higher energies led to more efficient spheroid dissection and reproducible results. Further increase in pulse energies (>9.1-9.2 μJ) caused the formation of a great number of cavitation bubbles and the chaotic displacement of the object from its initial position during laser exposure. As a result, the dissection of the spheroid chosen area could not be performed efficiently. Moreover, uncontrolled damage to the surrounding areas or spheroid might have occurred. Taking into account all the listed factors, the optimal energy of laser pulses was set in the range of 7-9 μJ, which corresponds to laser intensity of 1-1.2×10^10^ W/cm^2^. The process of laser exposure and formation of cavitation bubbles, leading to visible damage in spheroids is demonstrated in Fig. 2B,C, white arrow. As a result of exposure to laser radiation, a spontaneous opening of the wound edges was observed (Fig. 2; Movie 1). The schematic of spheroid obtaining and further laser exposure procedure, as well as indication of wound angular opening are presented in Fig. 2M.

**Fig. 3.**
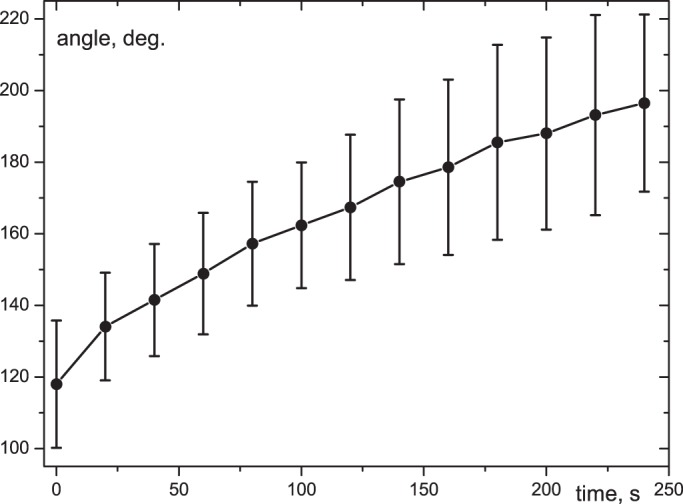
**Spontaneously increasing angular opening of the edges of the area damaged with nanosecond laser scalpel during the first four minutes after exposure.** The results of successive angle measurements of angular opening for 14 single spheroids, mean±s.d.

Correlation between the change of angular opening of the wound surface and time (from 0 to 240 s after laser impact) was evaluated for 14 single spheroids. Immediately after microdissection, the wound edges opened up to 118±18° and, in the subsequent four minutes, increased up to 197±25° (Fig. 3). The further observed growth of angular opening was negligible.

**Fig. 4.**
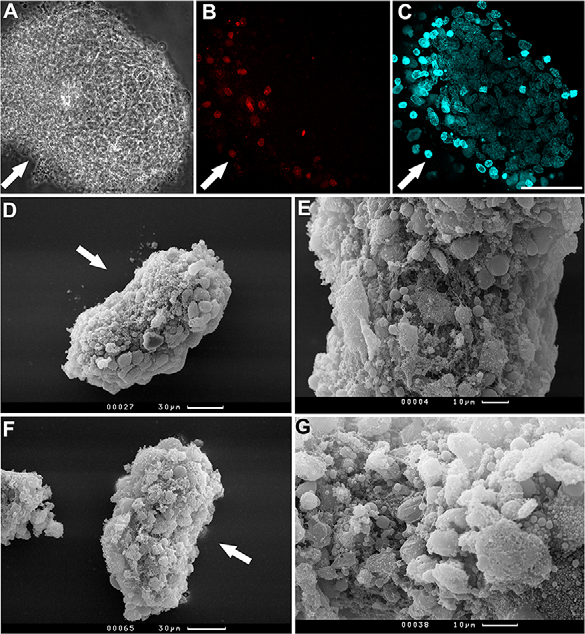
**Study of cell viability and the structure of BM MMSC spheroids surface after an injury with the nanosecond laser scalpel.** An hour after optimized laser exposure the wound area of spheroid (A) contained dead cells, stained with propidium iodide (B), while other cells remained viable, as indicated by the absence of propidium iodide in nuclei counterstained with Hoechst33342 (C). The rounded cells in the wound area were indicated at 30 min (D,E) and 1 h (F,G) after laser exposure. D,F, the general view of the spheroids; E, the enlarged centre; G, the enlarged edge of the wound to the right. Arrows indicate the wound area. A, phase contrast light microscopy; B,C, laser scanning confocal microscopy; D-G, scanning electron microscopy.

We observed the formation of fragments of dead cells in the area of injury within the first hour after laser exposure (Fig. 4A-C; Fig. S1). The increase of angular opening of the wound edges was accompanied by a change in morphology of the cells at the wound surface, with their shape changing from elongated and flattened to the round one. The structure of the surface layer in the intact area remained virtually unchanged and retained its integrity, while the cells remained flattened (Fig. 4D-G).

### Reparation of BM MMSC spheroids after the injury with nanosecond laser scalpel

**Fig. 5.**
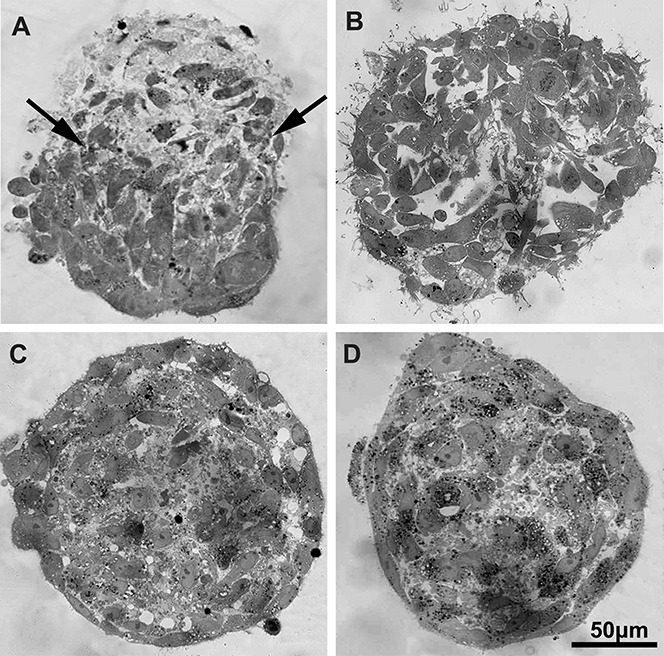
**BM MMSC spheroid structure repair after the exposure to nanosecond laser scalpel.** Damaged spheroids at 1 h after exposure had a clearly visible damaged area containing debris and a small number of rounded and polygonal cells (A). At 6 h, initial steps of integrity recovery were already taking place, so that wound edges became indistinguishable (B). At 24 h, the surface layer was closed (C) and by the seventh day the initial spheroid structure was fully recovered (D). Arrows indicate wound edges. Semi-fine sections, stained with methylene green, light microscopy.

The results of fluorescence microscopic, electron microscopic (Fig. 4) and histological analysis (Fig. 5) showed that, within the first 60 min after microdissection, wound surface contained fragments of dead cells and rounded cells (Fig. 4; Fig. 5A). In the area of injury, the original structure of spheroid was disrupted, and morphology of the surface and the inner zones became unified: i.e. the cells changed their shape and became polygonal or rounded. In uninjured regions of spheroids in the experimental group, the initial structure (surface area consisting of flattened cells and distinct from polygonal cells in the inner zone) were preserved (Fig. 5A). Directly in the area of damage, there were only single cells that maintained their original morphology; they were surrounded by cellular debris, cell surface was rough and cytoplasmic integrity was compromised.

**Fig. 6.**
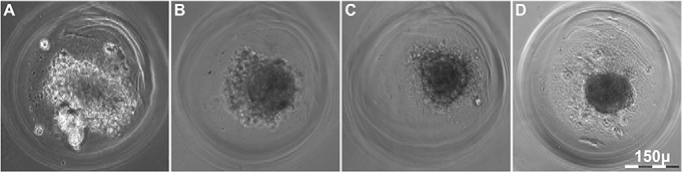
**Long live time-lapse microscopy during seven days after BM MMSC spheroid microdissection with nanosecond laser scalpel.** (A) 6 h, (B) 12 h, (C) 24 h and (D) 7 days after nanosecond laser scalpel impact. Light microscopy, phase contrast.

After six hours, the first steps of spheroid structure restoration were observed, but the surface cells in the area of injury remained polygonal (Fig. 5B). A day after the injury, spheroid structure was partially restored and cells in the surface layers began to flatten (Fig. 5C). By the third day, there was only one layer of flattened cells in spheroids of the experimental group, with the cells of polygonal shape still located deeper. Full restoration of the initial structure of the spheroids, with a few surface layers of flattened imbricated cells and polygonal cells of the inner zone, occurred seven days after microdissection (Fig. 5D). The described dynamics of repair were confirmed by the data obtained during live time-lapse microscopy of the experimental spheroids for seven days after laser microdissection (Fig. 6; Movie 2). Furthermore, immunocytochemical staining against Ki67 (the marker of cell proliferation) at 24 h after injury, when the initial wound closure was taking place, showed that there were no cells in active phases of cell cycle (Fig. S2), and it was also noted that the resulting diameter of spheroid after repair was less than the initial one (data not shown).

## DISCUSSION

Repair and restoration of tissues play an important role in the normal functioning of the body. Repair mechanisms and problems of their failure, such as scarring of the parenchymal organs and skin, are not fully understood. A long-surviving, simple, reproducible 3D cell system – i.e. spheroid – allows for the maintenance of the viable, functional and physiological state of cells in the absence of exogenous humoral and endocrine factors. Developing a model of spheroid damage using microdissection technique opens up the possibilities to study the mechanisms of regeneration and the role of spheroid-forming cells in repair.

Obtaining multi-cellular spherical aggregates from embryonic stem cells *in vitro* was described over 70 years earlier. To date, spheroids from embryonic stem cells, oncogenic lines, and transformed and primary cultures of different cell types have been obtained and characterized. Intercellular adhesion, cell-matrix adhesion, cytoskeletal and extracellular matrix-mediated factors act as basal factors to maintain the shape of spheroids in response to external forces, including gravity and hydrophobic-hydrophilic interactions. In addition, it is known that 3D cultures promote preservation and maintenance of the cells' ability in relation to differentiation and morphogenesis (Cesarz and Tamama, 2016). Under 3D conditions, cells retain the nuclear-cytoplasmic ratio and the ratio of the cytoplasm volume to the membrane area characteristic of *in vivo* conditions, which is required for successful functioning and communication (Baker and Chen, 2012), making cells more stable than in regularly passaged 2D monolayer cultures (Antoni et al., 2015). Different culture systems, such as ‘hanging drop’, nonadherent microplates, nonadherent and modified surfaces and rotary bioreactors, are used for obtaining cellular spheroids without the addition of the exogenous matrix (Benien and Swami, 2014; Kelm et al., 2003). Regardless of the technique used, mesenchymal cells from various sources are capable of forming viable dense spheroids under 3D conditions (Cesarz and Tamama, 2016; Repin et al., 2014; Zimmermann and McDevitt, 2014; Saburina et al., 2013).

Up to date, MMSC from adult bone marrow stroma are the best-characterized mesoderm-derived stromal cells with multipotent differentiation capacity. Moreover, MMSCs are known to possess strong immunomodulatory and anti-inflammatory properties and thus may contribute to tissue repair structurally and by modulation of local environment (Klimczak, Kozlowska, 2016).

It was shown that, after seven days in 3D culture, BM MMSC single cell suspension formed spheroids with a dense zone of flattened cells at the surface and irregular shaped central cells (Frith et al., 2010). In addition, the cultivation of BM MMSC under 3D conditions stimulated an active expression of mitogens, angiogenic and anti-inflammatory factors, and numerous cytokines (Cesarz and Tamama, 2016; Heo et al., 2016; Bartosh et al., 2010). Previous studies into the selection of optimal conditions for self-assembly of different types of cells in the 3D system influenced by gravitational forces and data about structure of obtained spheroids (Kosheleva et al., 2015; Repin et al., 2014) are consistent with the present data. The obtained seven-day BM MMSC spheroids (with 150-200 μm diameter) consisted of two to four layers of dense flattened surface cells and a loose inner zone containing polygonal cells and the extracellular matrix. This obtained and highly reproducible spheroid structure, resembling epithelial and mesenchymal tissue components, as well as described properties of BM MMSC cell population, led us to choosing these objects as a model for studying regeneration processes *in vitro*.

The developed model of laser microdissection and optimally chosen conditions of laser exposure allowed us to damage the surface and the inner area in a selected region of spheroid without disturbing the viability of the object in general. At the selected modes of laser irradiation, a lot of cell fragments appeared in the wound area of a spheroid, which is directly related to the physical effects, while a spheroid in the uninjured area retained the initial structure. The angular opening at the wound surface increased by more than 70° in the first four minutes after laser impact, which partially contributed to the rapid clearing of the wound from dead cells and their fragments. Such a change could indicate an artificial mechanical stress relaxation, which plays an important role in embryogenesis, morphogenesis and differentiation (Fouchard et al., 2014; Belousov et al., 1999). Spheroids are relatively soft, inelastic structures, in which the balance between cell-cell adhesion, cell-matrix adhesion, cytoskeletal and extracellular matrix-mediated factors may change as a function of time. In structures formed using scaffold-free, non-adherent systems, cells organize cortical cytoskeleton, which can dynamically change under different tensions (Czajka et al., 2014). In 30 min after microdissection, cells at the edges of the wound area acquired a rounded shape, which is probably due to loss of intercellular junctions and cytoskeleton reorganization. Mechanical stress relaxation caused by microdissection was likely to lower the surface tension and, therefore, contributed to the rapid changes in cell shape. The appearance of debris around the wound area could have appeared due to a partial loss of rounded cells caused by detachment-induced apoptosis – anoikis (Grossmann, 2002).

The sphere is the ‘default’ tissue morphology under non-adherent conditions, by having the smallest surface area per unit volume (cell). Minimal surface area translates into minimal interfacial tension and, therefore, the lowest energy requirement to maintain. Indeed, we observed a recovery of the spherical shape of spheroids in the first hours after injury. The internal structure of spheroids recovered gradually and, within seven days, cells in the irradiated area gradually acquired the initial morphology, the dense layered structure of the surface zone with flattened cells was restored and the repair processes were complete. We noted that spheroids contained no proliferating cells and the diameters of spheroids repaired after microdissection decreased compared to the initial ones, while the edges of the wound did not close, the defect seemed to be filled with undamaged cells of the inner zone (Movie 2). In this regard, we can assume that the repair of BM MMSC spheroids after microdissection is performed not as a result of cell proliferation, as in epimorphic regeneration, but due to the remodelling of existing viable cells.

### Conclusions

Studying regeneration *in vitro* using monolayer cell cultures allows us to evaluate only proliferation and migration of cells, and only in one plane. Hence, models accounting for 3D complex interaction of cells with each other and with the extracellular matrix are required for a detailed understanding of regeneration processes. *In vivo* experiments on animals represent one such model, but they are time-consuming, not always available, costly and poorly reproducible due to multiple factors. We have proposed a new simple reproducible model for studying regeneration *in vitro* in 3D cell spheroids. This system allows us to combine cells of different phenotype, origin, etc., to vary the supplements to the basal growth media, which, in combination with the innovative technology of laser microdissection, opens up opportunities for searching and exploring new ways to stimulate repair.

## MATERIALS AND METHODS

### 2D cell culture for cell spheroids technique

Primary cultures of human BM MMSC were used in the experiment. Samples of the bone marrow were obtained after voluntary written informed consent of patients. All the procedures were approved by the Ethical Committee of Federal State Budgetary Scientific Institution ‘Institute of General Pathology and Pathophysiology’, while performed in accordance with the Helsinki Declaration.

Cells were cultured onto Petri dishes under standard conditions (37°C, 5% CO_2_) in complete growth medium containing DMEM/F12 (1:1, BioloT, St. Petersburg), L-gluthamine (2 mM/l, PanEco, Moscow), gentamicin (50 μg/ml, PanEco, Moscow), insulin-transferrin-selenite (1:100, BioloT, St. Petersburg), 20 ng/ml bFGF (ProSpec, Israel) and 10% foetal bovine serum (HyClone, USA). The medium was replaced two to three times a week. Cell phenotype and culture confluence were controlled under an inverted light phase-contrast microscope CKX41 (Olympus, Japan), while photorecording was performed using the DeltaPix Viewer digital camera (Olympus, Japan). At 70% confluence, cells were passaged, with the fourth passage cultures used for immunophenotype analysis and spheroid formation.

Immunophenotyping of obtained cultures was performed using the following marker proteins: CD29, CD90, CD105, CD45, CD34, CD14, CD11b and CD19. For immunophenotype analysis, cells were washed from the complete growth medium with versene solution (BioloT, St. Petersburg), treated with 0.25% trypsin solution (BioloT, St. Petersburg), and the enzyme was then inactivated by addition of serum-containing medium, before cells were counted and centrifuged (7 min, 1000 ***g***). The resulting precipitate was resuspended and aliquoted in a solution of phosphate-buffer saline (pH 7.4) containing 1% of serum. Each sample was incubated in the dark (15 min, 25°C) with antibodies conjugated to fluorescent labels CD29-FITC (Beckman Coulter, 6604105, 1:100), CD90-PC5 (Beckman Coulter, IM3703, 1:100), CD105-PE (Beckman Coulter, AO7414, 1:100), CD45-FITC/CD14-PE (Beckman Coulter, IM1387, 1:100), CD34-FITC (Beckman Coulter, IM1870, 1:100), CD11b-FITC (Beckman Coulter, IM5030U, 1:100) and CD19-FITC (Beckman Coulter, AO7768, 1:100). After centrifugation (5 min, 400 ***g***), samples were re-suspended in 1 ml solution of phosphate-buffer saline (pH 7.4) containing 1% of serum in flow cytometry test tubes. The obtained results were analysed by the flow cytometer Cytomics FC-500 (Beckman Coulter, Inc, USA).

### 3D culture, obtaining cell spheroids

To obtain spheroids in 3D culture conditions, suspensions of characterized BM MMSCs at the fourth passage were placed in agarose plates with micro-wells (Microtissue, USA) at 250,000 cells/ml concentration in complete growth medium. Agarose plates were transferred to wells of 12-well plates and were cultured for seven days under standard conditions (37°С, 5% СО_2_).

### Laser dissector

Spheroids were irradiated using the Palm CombiSystem (Zeiss, Germany) nanosecond laser scalpel (wavelength 355 nm, repetition rate 100 Hz, pulse duration 2 ns, pulse energy was varied in the range of 7-9 μJ, the speed of motorized stage movement 120 µm/s). Laser radiation was focused on the objects using micro-objective Zeiss Fluar (10×, NA 0.5). The focused spot radius was estimated to be ∼3.3 µm. PALM RoboPro software was used to control laser scalpel.

### Nanosecond laser BM MMSC spheroids microsurgery

The surface and inner spheroid layers were injured with nanosecond laser pulses. Laser dissection of spheroid layers was performed by applying laser pulses along the straight-line path (from spheroid periphery to the centre) as defined by the operator in the PALM RoboPro software. The length of laser irradiated path was chosen for each spheroid individually and was set to be equal to its radius (typically, 75-100 µm). The laser processing of the defined straight-line path was repeated for five to eight times. Every next cycle was characterized by the axial (along *z*-axis) laser beam focus shift to provide the dissection of spheroid in three dimensions. The schematics of experiment is presented in Fig. 2M, and the process of microdissection is shown in Movie 1. The dynamics of spontaneous reparation after irradiation were observed using light time-lapse microscopy and, in the first hour after injury, the angles of wound opening were measured using AxioVision 4.8 software (Zeiss, Germany). The results were analysed using distribution-free analogue of repeated measures method, also known as the Fridman test.

A total of 25-30 spheroids were irradiated in every agarose plate, which took approximately 30 min. After irradiation, spheroids from experimental and control groups were returned under normal conditions in a СО_2_ incubator (+37°С, 5% СО_2_) for further observation of the wound healing process, or were fixed for histological studies. The design of the study, including the number of objects in experimental and intact groups as well as the different types of analysis methods used, are represented in Table 2. Spheroids were fixed at 30 min, 1 h, 6 h, 24 h, 3 days and 7 days after laser microsurgery.

**Table 2.**
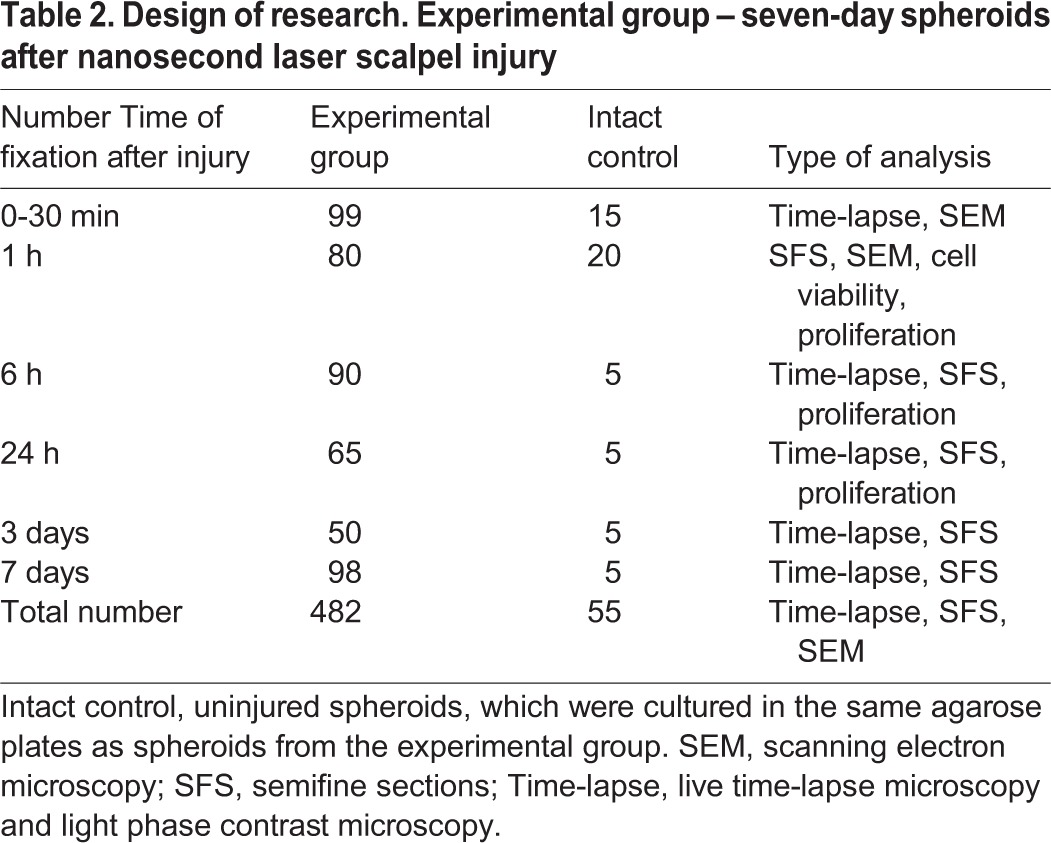
**Design of research. Experimental group – seven-day spheroids after nanosecond laser scalpel injury**

### Time-lapse microscopy

The first 60 min after laser microsurgery time-lapse, microscopy was performed using the PALM CombiSystem installation (Zeiss, Germany) with photorecording every five to 10 s for the first 5 min after irradiation and later every 5 min.

Long-term live registration was performed for seven days after laser microsurgery under standard conditions (37°С, 5% СО_2_) in the chamber of time-lapse system Cell-IQ (CM Technologies, Finland), with photorecording every 20 min using Cell-IQ Imagen software. Images obtained were later analysed using Cell-IQ Analyzer software.

### Light and scanning electron microscopy

Spheroids were fixed in glutaric aldehyde (1.5% solution in 0.1 M cacodylate buffer, pH 7.3; 1-2 h), postfixed in OsO_4_ (1% water solution, 1-2 h), dehydrated in ethanol with rising concentrations of 50%, 70% and 96% (two times for 5 min in each concentration), and acetone (three times for 10 min). Objects were then embedded in the mixture of epoxides Araldite М and Araldite Hardener (Sigma, USA) with the addition of catalyst DMP30 (Chimmed, Moscow) and plasticizer dibutylphthalate (Chimmed, Moscow). Resulting blocks were incubated under +60°С for 3-5 days to provide epoxide polymerization.

Semifine sections (1-2 μm) were obtained using ultra microtome Leica EM UC6 (Austria) and stained with methylene green (Sigma, USA) for 2-3 min. Specimens were observed under a light microscope Olympus ВХ51 (Olympus, Japan) using a Color View II photo camera, while software Cell F was used for photorecording.

To study spheroids under a scanning electron microscope, the fixed and dehydrated specimens were exposed to critical point drying, mounted on an object table and laid over with fine gold particles. The resulting replica was analysed under a CamScan (Japan) scanning electron microscope.

### Cell viability analysis of spheroids after injury

One hour after laser microdissection spheroids were stained with fluorescent dye bisbenzimide for nuclei staining (3.2 nM/ml, 15 min, 37°C, Hoechst 33342; Fluka, Switzerland) and with propidium iodide for vitality assay (1.7 μM/ml, 15 min, 37°C; ICN Biomedicals, USA). The same spheroids were then stained with a cell-permeant dye Calcein AM to detect viable cells (5 min, 25°C; Thermo Scientific, USA). The results of staining were immediately analysed in visible and UV light under the Palm CombiSystem (Zeiss, Germany) and the Olympus Fluoview FV10I laser confocal scanning microscope (Olympus).

### Immunocytochemical proliferation analysis of spheroids after injury

For evaluating cell proliferation activity, spheroids were fixed with 4% paraformaldehyde (20 min; +4°C) at 24 h after microdissection. Samples were washed from fixator with the solution of phosphate-buffer saline (pH 7.4). Spheroids were then incubated with the primary antibodies against Ki-67 (Abcam, USA, 1:500) and with species-specific FITC-conjugated secondary antibodies (0.1 µg/ml; Thermo Scientific, USA). The nuclei were post-stained with fluorescent dye bisbenzimide (3.2 nM/ml, 15 min, 37°C, Hoechst 33342; Fluka, Switzerland). The resulting samples were analysed under visible and ultraviolet irradiation using a FluoView FV10i confocal laser scanning microscope (Olympus, Japan).
